# Health Care Cybersecurity Challenges and Solutions Under the Climate of COVID-19: Scoping Review

**DOI:** 10.2196/21747

**Published:** 2021-04-20

**Authors:** Ying He, Aliyu Aliyu, Mark Evans, Cunjin Luo

**Affiliations:** 1 School of Computer Science University of Nottingham Nottingham United Kingdom; 2 School of Computer Science and Informatics De Montfort University Leicester United Kingdom; 3 School of Computer Science and Electronic Engineering University of Essex Colchester United Kingdom; 4 Key Lab of Medical Electrophysiology, Ministry of Education Institute of Cardiovascular Research Southwest Medical University Luzhou China

**Keywords:** health care, security incidents, root causes, cybersecurity challenges, cybersecurity solutions, COVID-19, pandemics

## Abstract

**Background:**

COVID-19 has challenged the resilience of the health care information system, which has affected our ability to achieve the global goal of health and well-being. The pandemic has resulted in a number of recent cyberattacks on hospitals, pharmaceutical companies, the US Department of Health and Human Services, the World Health Organization and its partners, and others.

**Objective:**

The aim of this review was to identify key cybersecurity challenges, solutions adapted by the health sector, and areas of improvement needed to counteract the recent increases in cyberattacks (eg, phishing campaigns and ransomware attacks), which have been used by attackers to exploit vulnerabilities in technology and people introduced through changes to working practices in response to the COVID-19 pandemic.

**Methods:**

A scoping review was conducted by searching two major scientific databases (PubMed and Scopus) using the search formula “(covid OR healthcare) AND cybersecurity.” Reports, news articles, and industry white papers were also included if they were related directly to previously published works, or if they were the only available sources at the time of writing. Only articles in English published in the last decade were included (ie, 2011-2020) in order to focus on current issues, challenges, and solutions.

**Results:**

We identified 9 main challenges in cybersecurity, 11 key solutions that health care organizations adapted to address these challenges, and 4 key areas that need to be strengthened in terms of cybersecurity capacity in the health sector. We also found that the most prominent and significant methods of cyberattacks that occurred during the pandemic were related to phishing, ransomware, distributed denial-of-service attacks, and malware.

**Conclusions:**

This scoping review identified the most impactful methods of cyberattacks that targeted the health sector during the COVID-19 pandemic, as well as the challenges in cybersecurity, solutions, and areas in need of improvement. We provided useful insights to the health sector on cybersecurity issues during the COVID-19 pandemic as well as other epidemics or pandemics that may materialize in the future.

## Introduction

### Background

COVID-19 has been an unprecedented challenge for the global health care system. It has further challenged the resilience of the health information system, which has affected our ability to achieve the global goal of health and well-being. The sector has become a primary target of adapted cybersecurity attacks [1,2]. To manage the pandemic and this extraordinary situation, the health sector has shifted its focus from the security of their systems and practices to their primary duty of delivering health care in order to save lives, placing themselves in a vulnerable situation. Attackers are taking advantage of the COVID-19 pandemic and have launched a number of cyberattacks against health care organizations [3-8]. Recent cyberattacks have impacted health care organizations such as Brno University Hospital [3], the US Department of Health and Human Services [4], the World Health Organization (WHO) [5], Gilead Sciences, Inc [6], hospitals in Romania [7], as well as the general supply chain of the health sector [8]. The health sector must be prepared to counteract cyberattacks in order to protect the availability of essential health care services as well as the confidentiality and integrity of health care information.

Cybercrime adapts to changes in the world situation very quickly. At the beginning of an escalation in the COVID-19 pandemic, malware cyberattackers identified common vulnerabilities and adapted their attacks to exploit these vulnerabilities. The current situation in the United Kingdom and worldwide provides a fertile breeding ground for various cyberattacks [9]. Cyberattackers are leveraging the increased reliance on remote working, decreased mobility, and the closure of borders between different countries, and the heightened demand for personal protective equipment (PPE) such as masks and gloves. The complex health care supply chain is also a target [10]. As a result, greater fear, uncertainty, and doubt is being experienced by the general population.

### Rationale

There is some research reviewing the literature on cybersecurity in the health sector. Jalali et al [11] performed a systematic review of the literature on cybersecurity response plans in health care. Coventry et al [12] conducted a narrative review on trends in cyber threats and ways forward in the health sector. Kruse et al [13] systematically reviewed health care–related cyber threats and trends. Offner et al [14] reviewed cyber threats and mitigation strategies among Australian health care organizations. Sardi et al [15] performed a systematic review of cyber risk in health facilities. However, there is limited research on an in-depth review and analysis of key cybersecurity challenges and solutions, specifically in the health sector, in the context of a pandemic situation such as COVID-19.

### Objective

Through a scoping review, this paper aims to identify the most prominent and significant methods of attack and threats that have affected the health sector during the COVID-19 pandemic, cybersecurity challenges, solutions, and areas that require further improvement. This research covers not only security-related matters as a result of the COVID-19 pandemic but also discusses inherent security challenges in health information systems that can be potentially exploited by attackers during the COVID-19 pandemic. It has implications for the whole spectrum of the health sector as a result of the increase in cybersecurity risks such as phishing, ransomware, and distributed denial-of-service (DDoS) attacks during the coronavirus crisis and in the long term.

## Methods

### Protocol and Registration

The review was performed according to the PRISMA-ScR (Preferred Reporting Items for Systematic Reviews and Meta-Analyses Extension for Scoping Reviews) checklist, proposed by the Joanna Briggs Institute [16]. The aim of this review is to identify health sector cyberattacks, security challenges, and solutions. Before undertaking this review, a protocol was created detailing sources of information, search strategies, eligibility criteria, source selection, and data charting processes. The PRISMA-ScR checklist is presented in Multimedia Appendix 1.

### Information Sources

A search of two major scientific databases (PubMed and Scopus) was performed to identify relevant articles. These include both original research articles and review articles.

### Search

The search formula “(covid OR healthcare) AND cybersecurity” was used to search for articles. The articles identified should have either a COVID-cybersecurity core or a healthcare–cybersecurity core.

### Eligibility Criteria

Only articles in English published in the last decade were included (ie, 2011-2020) in order to focus on current issues, challenges, and solutions. Reports, news articles, or websites were also included only when they are related directly to previously published work, or they were the only currently available information source at the time of manuscript preparation. Inclusion criteria were as follows: (1) relevance to health care cybersecurity and (2) coverage of well-discussed cybersecurity issues, challenges, and solutions.

### Selection of Sources of Evidence

**Figure 1:**
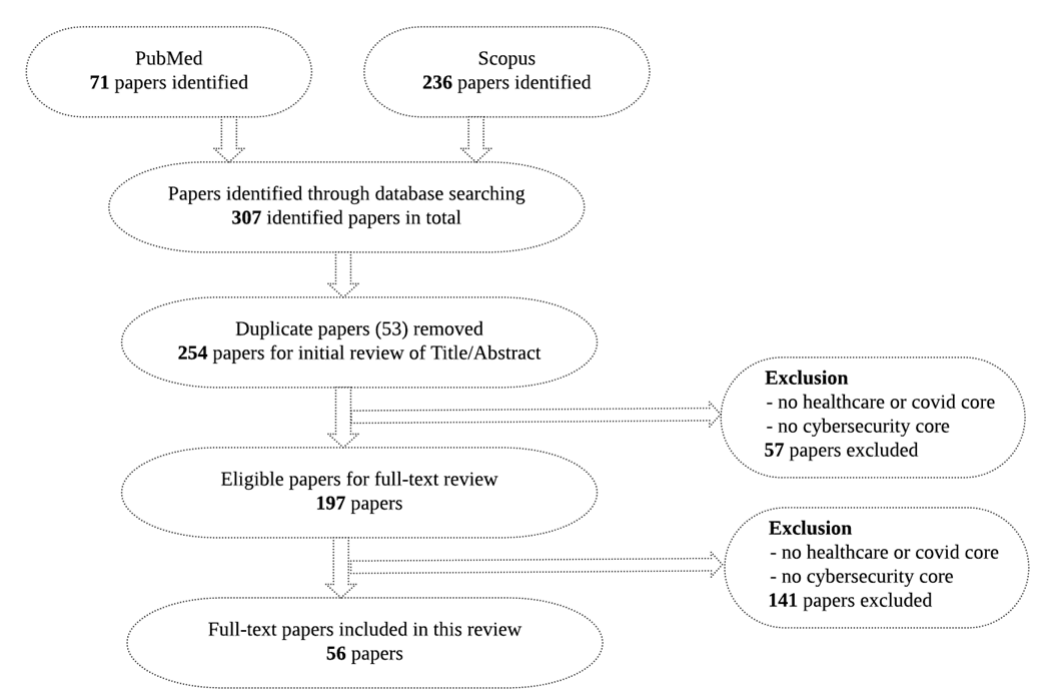
Flowchart showing the article identification and selection process.

The selection process is illustrated in Figure 1. The results of the search were exported to the EndNote library. The title and abstract of each paper were analyzed by 2 of the authors to assess eligibility. In cases in which this was not obvious, all 4 authors examined the paper and, when necessary, read it to assess relevance. A total of 307 identified papers were screened and 53 duplicates were removed. An additional 57 papers were excluded for not focusing on the healthcare–cybersecurity core or the COVID-cybersecurity core in the abstract. Another 197 papers were excluded for lacking these cores in the full text. In total, 56 papers were included in the review.

### Data Charting Process

The data were extracted and stored in a standardized Microsoft Excel (Microsoft Corp) form. This was an iterative process whereby the charting table is continually updated. Data charting was carried out both independently and collectively by at least two authors to ensure the quality of the extracted key findings from the literature before being used in the analysis.

### Data Items

Key data items, including title, abstract, authorship, aims, key findings related to the review objectives, evidence document, document type, year of publication, and location, were extracted.

### Critical Appraisal Within Sources of Evidence

Although the Joanna Briggs Institute suggests that the critical appraisal is usually not needed for a scoping review, we had at least 2 authors check the quality of the source of evidence to ensure they were relevant, up to date, and from reputable sources. In cases in which this was not obvious, all 4 authors assessed the sources.

### Synthesis of Results

By aggregating information from the selected literature, the results were analyzed and qualitatively presented in both tabular and descriptive formats (grouped into themes), which aligned with the objective and scope of the review.

## Results

Four themes were observed across the selected literature: (1) health sector condition changes due to COVID-19, (2) health care cyberattacks during the COVID-19 pandemic, (3) health care cybersecurity challenges, and (4) health care cybersecurity controls.

### Health Sector Condition Changes Due to COVID-19

The findings pertaining to changes in conditions in the health sector as a result of COVID-19 are summarized in Table 1. The main changes to health services caused by the COVID-19 pandemic include decreased mobility, border closures, and the increasing reliance on remote work, often carried out with little previous experience and planning. These conditions have made the health sector more vulnerable to potential cyberattacks [1,2,17].

**Table 1:** Health sector condition changes due to COVID-19.

Changes	Reference
The decreased mobility and border closures, and the increasing reliance on remote work, create challenges to health sector	Hakak et al [1], Williams et al [2], Schneck [17]
New technologies such as eConsultation services for patients and electronic multidisciplinary teams leave users open to a variety of attacks	Weil and Murugesan [18]
Health service staff often have limited experience in working remotely, leaving the sector vulnerable to cyberattacks, such as malwares	Boddy et al [9], Offner et al [14], Jalali et al [19], Hoffman [20], Ronquillo et al [21]
The health care industry significantly lags behind other industries in terms of cybersecurity and digital literacy is lacking among staff working from home, making it a prominent target	Sardi et al [15], Kim et al [22]
The increase in demand for certain goods such as PPE^a^ makes health services and governments exposed to digital scams such as luring emails with the intention of stealing sensitive information	Schneck [17]

^a^PPE: personal protective equipment.

As health staff and patients are restricted in terms of movement due to the lockdown, the decrease in mobility and border closures make individuals and organizations turn to technology to provide essential health services such as appointments, diagnosis, and even operations. Examples are the use of eConsultation (electronic consultation) services for patients and electronic multidisciplinary teams. Although these technologies have their advantages, they leave users and receivers of these technologies open to a variety of attacks such as phishing campaigns and ransomware attacks [18].

Furthermore, health services staff often have limited previous experience with remote working and with planning for this change, which leaves the sector vulnerable to cyberattacks [9,14,19]. As health services make use of a variety of medical devices, interconnectivity and interoperability create issues as they are now being accessed from outside health services’ internal network perimeter. The medium and mode of access creates problems as access to the sensitive parts of health services can be reached via unsecured network connections or unpatched systems by staff working remotely [19]. In addition, some medical devices use off-the-shelf software, such as commercial operating systems (eg, older versions of Windows). These systems are vulnerable to a large variety of threats such as malware, ransomware, etc [20,21]. Overall, the health care industry significantly lags behind other industries in terms of cybersecurity and coupled with a lack of digital literacy among staff mostly working from home, makes it a prominent target [15,22].

Additionally, the increase in demand for certain goods such as PPE and other protective merchandise such as masks, gloves, etc, are exposing health services and even governments to digital scams, especially in the form of phishing attacks. As health services are in need of these essential items, they can be targeted by adversaries via luring emails with the intention of stealing sensitive information [17].

### Health Care Cyber Attacks During the COVID-19 Pandemic

Multiple cyberattacks occurred at the beginning of the global COVID-19 pandemic (early 2020) in the health sector. We selected well-documented cyberattacks with detailed information available, including root causes and consequences. The main findings are summarized in Table 2.

**Table 2:** Security incidents during the COVID-19 pandemic.

Security incidents	Type of attack	Impact
Brno University Hospital [3]	Ransomware	Postponement of surgeries, appointments, etc
US Department of Health and Human Services [4]	Distributed denial of service	Disruption to COVID-19 pandemic responses
World Health Organization [5]	Ransomware/phishing	Defacement and misinformation
Gilead Sciences, Inc [6]	Phishing	Impersonation and exfiltration
Hospitals in Romania [7]	Phishing/ransomware	Disruption and exfiltration
Health care supply chains [8]	Malware	Disruption of activities

Brno University Hospital in the Czech Republic, which is one of the country’s main COVID-19 testing centers, was struck by ransomware, resulting in the postponement of surgeries. The ransomware infection was confirmed in the early hours of the day when the hospital decided to disconnect all computer networks. It was noticed that the ransomware infection was gradually replicating, and all the individual systems were failing. As a result, all computers had to be shut down. The hospital is reported to be still recovering capabilities, as it is not yet fully operational due to the attack [3]. The attack had an impact on the activities of the hospital as there was no database systems, that is, means of storing data; hence, staff have had to write and transfer their notes manually. This leads to slow processes and can potentially endanger lives in these trying times.

The US Department of Health and Human Services experienced a DDoS attack intended to disrupt the organization’s responses to the COVID-19 pandemic. This attack targeted its servers by overloading it with millions of hits over several hours [4]. It was reported as a campaign of disruption aimed at hindering the response to the coronavirus pandemic as the targeted agency was tasked with protecting the health of citizens and delivering essential human services. Although the agency claimed the attack was not successful, and that the attackers did not infiltrate the internal network nor steal any data, this demonstrates that attacks like these can cause damage not just to the services of health agencies but also to the lives that depend on it, especially in times of emergencies.

Increased phishing website hacking attempts on the WHO and its partners led to the WHO putting out a warning to the general public to be more careful [5], as it has been reported that over 4000 coronavirus-related domains (ie, domains that contain words like “corona” or “covid”) have been registered since the beginning of 2020. These registered domains were used by adversaries for phishing-related activities. Thus, the WHO incident was orchestrated by hackers in order to steal passwords. It was reported that a group of hackers created a malicious website posing as an email login portal for WHO employees in an attempt to steal their passwords. Although the WHO claims the attack was not successful, it still shows that phishing attacks can be leveraged to target health organizations.

Coronavirus vaccine manufacturer Gilead Sciences, Inc, was also targeted by hackers [6]. Staff at this pharmaceutical company were targeted via a fake email login page that was designed to steal passwords. It was reported that the attack was an attempt to compromise the email accounts of staff at the company using messages that impersonated journalists.

Hospitals in Romania experienced ransomware attacks by hackers as well [7]. The hackers were planning to use COVID-19–themed emails to infect these hospitals with ransomware. Their motivation was the protest against the COVID-19 quarantine measures of the country. The hackers owned malwares (eg, remote access trojans, ransomware, website defacements, and SQL injection tools) that can be used to bring down servers and steal information. It was reported that they intended to send emails about COVID-19 to hospitals to infect computers, encrypt files, and disrupt hospital activities. However, the attack was not as successful as the hackers were tracked down and arrested by Romanian law enforcement.

It has been reported that Interpol has cautioned agencies around the world about a significant rise in the global number of ransomware attacks explicitly targeting hospitals and health institutions [8]. It discovered that there was an increase in the number of attempted ransomware attacks on organizations in the 194 member countries. Additionally, a cyber warning was issued for key health care organizations involved in the coronavirus response both in the United Kingdom and the United States. A joint statement by the United Kingdom’s National Cyber Security Centre (NCSC) and US Cybersecurity and Infrastructure Security Agency revealed that malicious cyber campaigns had been uncovered, with large-scale “password spraying” campaigns directed at health care bodies and medical research organizations in both nations [23].

Health care supply chains have not been omitted from these attacks; the US Federal Bureau of Investigation (FBI) issued a warning about a malware targeting this sector. The malware is called Kwampirs, a remote access Trojan that exploits network vulnerabilities of targeted organizations across the United States, Europe, Asia, and the Middle East [24]. The infected supply chain components included cyber-physical systems assets in health care organizations. The FBI alerted the health care sector against future cyberattacks, as Kwampirs have been historically targeting health care organizations.

The analysis of the above-mentioned incidents indicate that the health sector has become a primary target of cybersecurity attacks. Attackers are taking advantage of the COVID-19 pandemic and launching attacks, which are mainly ransomware, DDoS, phishing, and other type of malwares. The health care supply chain can be more vulnerable to cyberattacks especially during pandemics. The cyberattacks have resulted in negative impacts on the availability of essential health care services and challenged health care organizations in the protection of the confidentiality and integrity of health care information.

### Health Care Cybersecurity Challenges

Selected papers discussing the main challenges of cybersecurity in the health sector were reviewed, and the main findings are summarized in Table 3.

**Table 3:** Key health sector security challenges and associated vulnerabilities.

Key challenges and published vulnerabilities		Reference
**Remote work security assurance**		
	There are known security vulnerabilities with remote desktop protocols and virtual private networks	Argaw et al [10]
	There are known attacks on health care system such as distributed denial-of-service attacks, malware, etc	Offner et al [14]
	Cyberattacks target innumerable wireless connected devices in health care	Boddy et al [9]
**Endpoint device management**		
	An endpoint device can provide an entry point to larger health care networks	Coventry et al [12]
	The integration of new endpoint devices with outdated, legacy, or unsupported operating systems compromises interoperability and increases cybersecurity vulnerability	Kruse et al [13], Naidoo [25]
	The health sector relies heavily on perimeter defense (antivirus, firewalls) for protection against cyber risk	Reagin and Gentry [26]
	The factor that most influences cybersecurity in a hospital is endpoint complexity	Jalali and Kaiser [27]
**Human factors in cybersecurity**		
	The majority of information security incidents are related to human error	Evans et al [28], Evans et al [29]
	There is a statistically significant positive correlation between workload and the probability of health care staff opening a phishing email	Jalali et al [19]
	The health sector lacks root cause analysis and cybersecurity incident prevention, especially those through unintentional human error	Evans et al [28], Evans et al [29]
	Although some effort has been made to analyze human error (eg, use of IS-CHEC^a^), such approaches have not been widely adopted	Evans et al [30]
**Lack of security awareness**		
	There is low awareness in the health sector of cyber risks	Gordon et al [31]
	The most common action taken in response to breaches or attacks is additional staff training or communication	Furnell and Shah [32]
	Health staff has poor awareness of consequences of behavior, and there is a lack of policies and reinforcement of secure behavior	Coventry et al [33]
	There is a lack of pandemic-specific cybersecurity training campaigns, documented procedures, and guidance on revised procedures and technologies	Kaplan [34]
**Inadequate board-level risk assessment communication**		
	There is a need for a matrix that can translate the strategic requirements of a health care system into prioritized cyber improvement needs	Barad [35]
	There is a lack of understanding of security risks and its impact on organization-wide risk management	Tully et al [36]
	There is a lack of appreciation among health care executive management of the business risk impact associated with cyber breaches	Jones and Katzis [37]
**Inadequate business continuity plans**		
	Risks will continue to grow if cybersecurity is not designed into the product from the beginning of the product or project life cycle	Coventry and Branley [12]
	The key security risks challenging business continuity are vendor dependence, inappropriate encryption configurations, and the inability to handle health information sharing and exchange with third-party and cross-border partners	Frontoni et al [38], Bhatia and Ibrahim [39], Natsiavas et al [40], Nalin et al [41]
	The health sector lacks sophisticated data security tools compared to other industries	Walker-Roberts et al [42]
	Cybersecurity capability is a strategic asset that every health organization must adopt, along with the concepts of building organizational resilience and the capacity to learn from mistakes	Jalali et al [11], Reagin and Gentry [26]
**Lack of coordinated incident response**		
	The health sector tends to have a time lag between an attack occurring and detection of the breach	Coventry and Branley [12]
	Current health care cyber defense is often reactive and undertaken after malicious attacks	Akinsanya et al [43]
	There is a lack of a coordinated incident response capacity to actively counteract constantly emerging and evolving malware threats	Chen et al [44]
	Cybersecurity should be a team effort, from board members to front-line employees, with all being held accountable for cybersecurity	Pullin [45]
**Limited budget and the need to deliver health care services without disruption**		
	There is a lack of experienced cybersecurity experts in the health care industry	Argaw et al [46]
	There is a lack of a value-based system to weigh and balance benefits and risks in aspects of security, privacy, and adoption of technology	Boddy et al [9]
**Vulnerable MCPS^b^**		
	Limited MCPS capability makes the health sector vulnerable to compromises	Almohri et al [47]
	The reliance on the health care network increases cybersecurity risks to health care systems	Zheng et al [48]
	Cyber threats can be introduced to the MCPS though vulnerable IoT^c^ devices	Jimenez et al [49]

^a^IS-CHEC: Information Security Core Human Error Causes.

^b^MCPS: medical cyber-physical systems.

^c^IoT: internet of things.

The analysis shows that the main cybersecurity challenges of the health sector are remote work security assurance, endpoint device management, human errors, the lack of security awareness, inadequate senior-level security risk assessment, inadequate business continuity plans, the lack of coordinated incident response, constrains on budget and resources, and vulnerability of medical systems. These challenges cover not only the security-related matters as a result of the COVID-19 pandemic but also the inherent security challenges in the health sector that can be potentially exploited by attackers during the COVID-19 pandemic. It is imperative for the health care organizations to identify these challenges and take actions for prevention.

#### Remote Working Security Assurance

As remote working is now an integral element of health care service delivery, health staff are relying on enterprise remote desktop protocols and virtual private networks (VPN) to access internal networks. However, these come with certain risks that adversaries are looking to exploit. For example, the remote desktop protocol has a history of security issues and generally should not be publicly accessible without additional protections such as firewall, whitelist, and multifactor authentication [10]. Likewise, VPNs also have some known and unknown vulnerabilities, both on the client and server side, which have been exploited for years by cybercriminals [19]. The DDoS attacks on health care systems [14] and the innumerable wireless connected devices [9] have created further challenges to a remote work environment.

#### Endpoint Device Management

A number of endpoint devices, which comprises various patient-monitoring equipment that either connects to the internet or legacy-dispersed networks, are often unpatched [12]. This risk further increased during the pandemic as a result of organizations competing to procure internet of things (IoT) devices during the COVID-19 pandemic for their staff, which resulted in more employees than before using personal devices to perform work from home. From an enterprise architecture perspective, having tighter integration across the information technology (IT) environment is positive in terms of the organization being more agile; however, it makes the network vulnerable to cyberattacks such as email phishing, ransomware, DDoS, and network data breaches [13]. The integration of new endpoint devices with outdated legacy systems can increase vulnerabilities [13,25]. However, organizations overly rely on perimeter defense (antivirus, firewalls) and other forms of basic protection against cyberattacks [26]. By interviewing 19 C-Suite cybersecurity professionals, Jalali et al [27] also confirmed the factor that most influences cybersecurity in a hospital setting is endpoint complexity.

#### Human Factors in Cybersecurity

Existing research has shown that the majority of information security incidents are related to human error [28]. There is a tendency for human error when staff are busy focusing on saving lives and adjusting to new work environments and technologies. With sudden changes in working practices, being under stress for an extended period of time makes employees vulnerable to falling into malicious trickery and making mistakes [28]. According to Jalali at al [19], there is a statistically significant positive correlation between workload and the probability of a health care staff opening a phishing email. Naidoo et al [25] developed a multilevel influence model to explore how cybercriminals exploited the COVID-19 pandemic using social engineering techniques. However, the health sector lacks root cause analysis [28] to prevent human error related security incidents, especially those through unintentional human error [29]. Although some efforts have been made in applying the human reliability analysis technique in the context of information security (eg, Information Security Core Human Error Causes [IS-CHEC] [30]) to analyze human error, such approaches have not been widely adopted.

#### Lack of Security Awareness

Cybercriminals are exploiting people’s anxieties during the COVID-19 pandemic. Gordon et al [31] identified that there is low awareness in the health sector of risks. Furnell et al [32] identified that the most common action taken in response to the most disruptive breaches or attacks is additional staff training or communication. Coventry et al [33] reported that health staff had poor awareness of the consequences of certain behaviors, and there is a lack of policies and reinforcement of secure behavior. However, increased cybersecurity awareness is required for the health sector to protect themselves and their patients from potential cyber threats such as phishing and ransomware. Due to the lack of prior planning and training to work under pandemic situations, health care staff require more training and support, such as pandemic-specific cybersecurity training campaigns, documented procedures, and guidance on revised procedures and technologies [34]. For example, health sector staff should be made aware of and able to flag phishing emails containing buzzwords during a pandemic, such as “WHO” or “donation.” They should also be advised on how to validate trustworthy information sources in order to avoid ransomware attacks [1].

#### Inadequate Board-Level Risk Assessment Communication

There is a lack of understanding of security risks and its impact on organization-wide risk management, such as impacts on patient care and clinical outcomes [36]. The health sector lacks a matrix that can translate the strategic improvement needs of a health care system into prioritized information/cyber improvement needs [35]. Schwartz et al [37] identified that there is a lack of appreciation among health care executive management staff of the business risk impacts of cyber breaches.

#### Inadequate Business Continuity Plans

The health sector does not have enough data protection mechanisms; Walker-Roberts et al [42] confirmed that the health sector lacks sophisticated data security tools compared to other industries. Security is not built into its supply-chain and third-party vendors. Existing research shows that the key security risks challenging business continuity are vendor dependence, inappropriate encryption configurations, and the inability to handle health information sharing and exchange with third-party and cross-border partners [38-41]. Risks will continue to grow if cybersecurity is not integrated into the project life cycle from the beginning [12]. Cybersecurity capability is a strategic asset that every health organization must adopt, along with the concepts of building organizational resilience and the capacity to recover from incidents and learn from mistakes in order to maintain business continuity [11].

#### Lack of Coordinated Incident Response Involving Different Parties

As highlighted by Coventry and Branley [12], the health care sector has a exhibited a trend of having a time lag between the occurrence of an attack and its detection. In fact, this aids attackers by giving them more time to explore the network and conduct lateral movement, which increases the damage inflicted by security breaches. Current health care cyber defense response is often reactive and undertaken after malicious attacks [43], lacking a coordinated incident response capacity to counteract constantly emerging and evolving malware threats [44]. The failure of health care organizations in having a successful and secure backup mechanism in place makes it frail in terms of incident response and recovery [12]. Pullin et al [45] also confirmed that cybersecurity should be a team effort, with everyone from board members to front-line employees being held accountable for cybersecurity.

#### Limited Budget and the Need to Deliver Health Care Services Without Disruption

Although health care services are spending funds to become more integrated to deliver health care services without disruption [9], the necessary emphasis is not given to the security aspect in terms of upkeep (eg, keeping software updated and systems secure). However, this is reported to be due to a shortage in experienced cybersecurity experts within health care organizations with the required skills and experience to enable health care organizations to change their business operations at significant pace without undertaking the “usual” levels of cybersecurity assurance [46]. Boddy et al [9] identified the needs of a value-based system to weigh and balance the benefits and risks in aspects of security, privacy, and adoption of technology.

#### Vulnerable Medical Cyber-Physical Systems

Cybersecurity measures such as vulnerability scans or patch management are often not available or only possible by manufacturers [49]. Their basic limited capability makes them vulnerable to compromise [47]. Cybersecurity measures such as vulnerability scans or patch management are often not available or only accessible for manufacturers. Moreover, their connection and reliance upon the health care network significantly increase the cybersecurity risk to the entire health care system [48]. With the widespread use of IoT medical devices, cyber threats can be introduced to medical cyber-physical systems though vulnerable IoT devices [44].

### Health Care Cybersecurity Controls

Selected papers discussing cybersecurity solutions present within the health sector were reviewed, and the main findings are summarized in Table 4.

**Table 4:** Crucial health sector security solutions.

Solution		Reference
**Apply endpoint device management tools**		
	Apply perimeter-based defense (antivirus, firewalls) for protection against cyberattacks	Reagin and Gentry [26]
	Restrict the technologies and devices used by health staff to remain compliant with security regulations such as HIPAA^a^ during pandemics	Hoffman [20]
	Adapt the NIST^b^ approach to manage security IoT^c^ medical devices	Kelly et al [50]
**Secure the remote work environment**		
	Apply multifactor authentication	Argaw et al [10]
	Apply a chaotic map–based authenticated security framework for remote point of care	Deebak et al [51]
	Apply remote access monitoring such as the NHS^d^ attack surface reduction rules	Zorz [52]
	Apply perimeter security solution such as NHS Secure Boundary to enable secure access	NHS Digital [53]
	The health care sector needs to ensure data protection mechanisms for securing system access and transmitting data	Rezaeibagha et al [54]
**Raise security awareness**		
	Apply a holistic, integrated approach to improve staff awareness, competence, and mitigation of threats	Pullin [45], Sedlack [55]
	Implement cybersecurity training programs and cybersecurity awareness campaigns	Gordon et al [56]
	Apply the NCSC’s^e^ Board Toolkit to raise board-level security awareness	NHS Digital [57]
	Provide comprehensive employee training and education to enable the identification and assessment of risks	Alzahrani [58]
	Implement a positive organizational climate to influence people’s behavior	Kessler et al [59]
**Ensure business continuity**		
	Apply a self-assessment tool such as the NHS Data Security and Protection Toolkit	NHS Digital [60]
	Embrace cybersecurity and a develop strong culture of cyber vigilance	Dameff et al [61]
	Ensure business continuity through data backups, intrusion detection, and prevention systems	Rezaeibagha et al [54]
	Apply a systematic risk assessment of the impacts on health care business operations	Kim et al [22]
	Consider cybersecurity insurance in health care	Kabir et al [62]
**Apply technical controls**		
	Apply network segmentation to isolate network traffic	Hakak et al [1]
	Apply general technical controls including encryption, authentication, and authorization	Yaseen et al [63]
	Apply homomorphic encryption that ensures strong security and privacy guarantees while enabling analysis of encrypted data and sensitive medical information	Raisaro et al [64]
	Apply blockchain to facilitate health care interoperability	Narikimilli et al [65]
	Apply cryptographic security to address data sharing and storage of patient information across network systems	Pussewalage and Oleshchuk [66]
**Policies and legislations**		
	Laws and regulations can help to combat the issues of medical cyber-physical systems	Raisaro et al [64]
	Security instructions and control designs should be tailored	Wang and Jones [67]
	Regulatory changes or manufacturers should become more security-minded in the medical device design phase	Department of Health and Social Care, UK Government [68]
	Policymakers may need to alter policies to allow new technological innovations to be applied to health care	Bhuyan et al [69]
	The US Congress passed the 21st Century Cures Act to promote patient control over their own health information while protecting privacy and cybersecurity	Hoffman [20]
**Incident reporting and cyber threat intelligence support**		
	NHS Digital issued two high-severity CareCERT alerts (BlueKeep and DejaBlue) and developed a high-severity alert process handbook to facilitate incident reporting and sharing	Department of Health and Social Care, UK Government [68]
	Apply an evidence-based approach, such as the generic security template, for incident reporting and exchange	He and Johnson [70], He and Johnson [71]
	Establish an international workforce to facilitate cyber threat reporting and exchange to combat pandemic-themed cyber threats	Hakak et al [1]
**Cybersecurity guidance specific to COVID-19**		
	The NHS has added guidance on working from home securely in the context of COVID-19	NHS Digital [72]
	The United Kingdom’s Information Commissioner’s Office created an information hub to assist individuals and organizations to manage data protection during the COVID-19 pandemic	Information Commissioner’s Office [73]

^a^HIPAA: Health Insurance Portability and Accountability Act.

^b^NIST: National Institute of Standards and Technology.

^c^IoT: internet of things.

^d^NHS: National Health Service.

^e^NCSC: National Cyber Security Centre.

#### Apply Endpoint Device Protection

During the COVID-19 pandemic, health staff working from home may adopt telehealth technologies or IoT devices. This increases cybersecurity risks, as it expands the footprint for cyberattack to the use of new devices outside of the service providers’ network [50]. Health staff are advised to restrict the technologies and devices they used to remain compliant with security regulations such as Health Insurance Portability and Accountability Act during the pandemics [20]. However, health care organizations mainly reply on perimeter defense (eg, antivirus, firewalls) for protection against the potential cyberattacks [26]. The National Institute of Standards and Technology (NIST) has recently released a draft security guide and recommendations for managing the security IoT devices, but it is unclear whether it will be enforced across the health sector [50].

#### Secure Remote Work Environment

Existing solutions include the use of multifactor authentication and the monitoring of the log activity of user accounts and revoking account access if no longer needed [10]. Deebak et al [51] proposed a chaotic map–based authenticated security framework for remote point of care. Health organizations such as those in the United Kingdom have started using services to monitor their remote access infrastructure constantly and to investigate anomalies. For example, the National Health Service (NHS) has employed attack surface reduction rules (eg, block macros, executable content, process creation) [52]. Furthermore, a more recent NHS Digital service, Secure Boundary, was introduced as a perimeter security solution to enable secure access for NHS staff and to provide security monitoring [53].

#### Raise Security Awareness

Health care organizations already have cybersecurity programs in place to increase levels of security awareness [45,55]. Existing solutions include the use of cybersecurity training programs and cybersecurity awareness campaigns [56]. In a cybersecurity campaign, the IT department sends out fake phishing emails to their staff and provides further training to those who fail to identify these emails [56]. In the United Kingdom, more than 100 NHS boards have completed cybersecurity training accredited by the Government Communications Headquarters since the WannaCry attack. Furthermore, the NCSC’s Board Toolkit for the NHS provides additional information on ransomware and backups. NHS Digital also runs a cyber awareness campaign called the Keep I.T. Confidential campaign. Over 340 organizations have downloaded the materials since its launch in September 2019 [57]. However, there is not enough work on training programs tailored to the pandemic such as COVID-19–themed social engineering, although the world is realizing the importance of raising the awareness of COVID-19–related cyberattacks [58]. Existing research shows that positive organizational climate can influence people’s behavior [59].

#### Ensure Business Continuity

Health care leadership must embrace cybersecurity and develop strong cultures of cybervigilance [61]. The health sector already has business continuity solutions in place such as data backups and intrusion detection and prevention systems [54]. NHS trusts have been asked to follow and meet the Cyber Essentials and government standards. NHS Digital has launched a Data Security and Protection Toolkit [60], a self-assessment tool for organizations that need to access NHS patient information and systems. The toolkit must be applied to ensure that organizations practice good cyber hygiene. Security risk assessment is essential to ensure business continuity. Kim et al [22] systematically assessed the impacts of cybersecurity threats on remote health care. Cybersecurity insurance in health care [62] should also be considered as a solution to ensure business continuity management, but it has not been widely adopted.

#### Apply Technical Controls

General technical controls applied by the health sector include encryption, authentication, and authorization to protect data from cyber threats [63]. Cryptographic security is used to address data sharing and storage of patient information across network systems [66]. Homomorphic encryption is applied to ensure robust security and privacy guarantees while enabling analysis of encrypted data and sensitive medical information [64]. Blockchain is also applied to facilitate health care interoperability due to its immutability, transparency, and decentralization [65]. Network segmentation and isolation also need to be considered by the health sector [1]. With network segmentation, network traffic can be isolated and/or filtered to limit and/or prevent access between network zones. For example, in case of systems compromise, one should freeze any activity in the system, disconnect the infected machines from any external drive or medical device, and go offline from the network.

#### Policy and Legislation

The health sector already has security policies and legislation in place for cybersecurity management. Laws and regulations are available to protect medical cyber-physical systems [64]. Security controls need to be tailored according to regulation [67]. Manufacturers are also required to consider these regulations to design medical devices [68]. However, policymakers may need to alter policies to allow new technological innovations to be applied to health care [69]. The US Congress passed the 21st Century Cures Act to promote the interoperability of electronic health records and promote more patient control over one’s own health information while protecting privacy and cybersecurity [20]. However, more efforts are needed on security policies or legislations in handling cybersecurity-related matters during pandemics like COVID-19.

#### Incident Reporting and Cyber Threat Intelligence Support

The health sector is required to report cybersecurity incidents to a supervisory authority, such as the national Computer Security Incident Response Team in the European Union. In the United Kingdom, there is government-approved support from the NCSC. NHS Digital has issued two high-severity CareCERT alerts in 2019 (BlueKeep and DejaBlue). After developing a high-severity alert process handbook, remediation went from 18 weeks for BlueKeep down to 3 weeks for DejaBlue [68]. He and Johnson [70,71] proposed a generic security template, which is an evidence-based argumentation approach to facilitate incident reporting and exchange. This approach was applied to a health care organization but has not been widely adopted. Hakak et al [1] identified the needs of establishing an international workforce to facilitate threat reporting and cyber threat intelligence (eg, attack vectors and countermeasures) exchange to combat pandemic-themed cyber threats. The health sector will benefit from such practices during pandemics in order to avoid similar incidents.

#### Cybersecurity Guidance Specific to COVID-19

Some health care organizations have started providing security guidance specific to COVID-19 for their staff. For example, NHS Digital has added guidance on working from home security, ramping up its on-site support for trusts on risk mitigations, data backup, and threat response. They also offer the NHS the NCSC’s Protective Domain Name Service free of charge [72]. Furthermore, governments also provide cybersecurity guidance to both individuals and organizations. For example, the United Kingdom’s Information Commissioner’s Office created an information hub in order to assist individuals and organizations to protect data during the COVID-19 pandemic [73].

## Discussion

### Summary of Evidence

**Figure 2:**
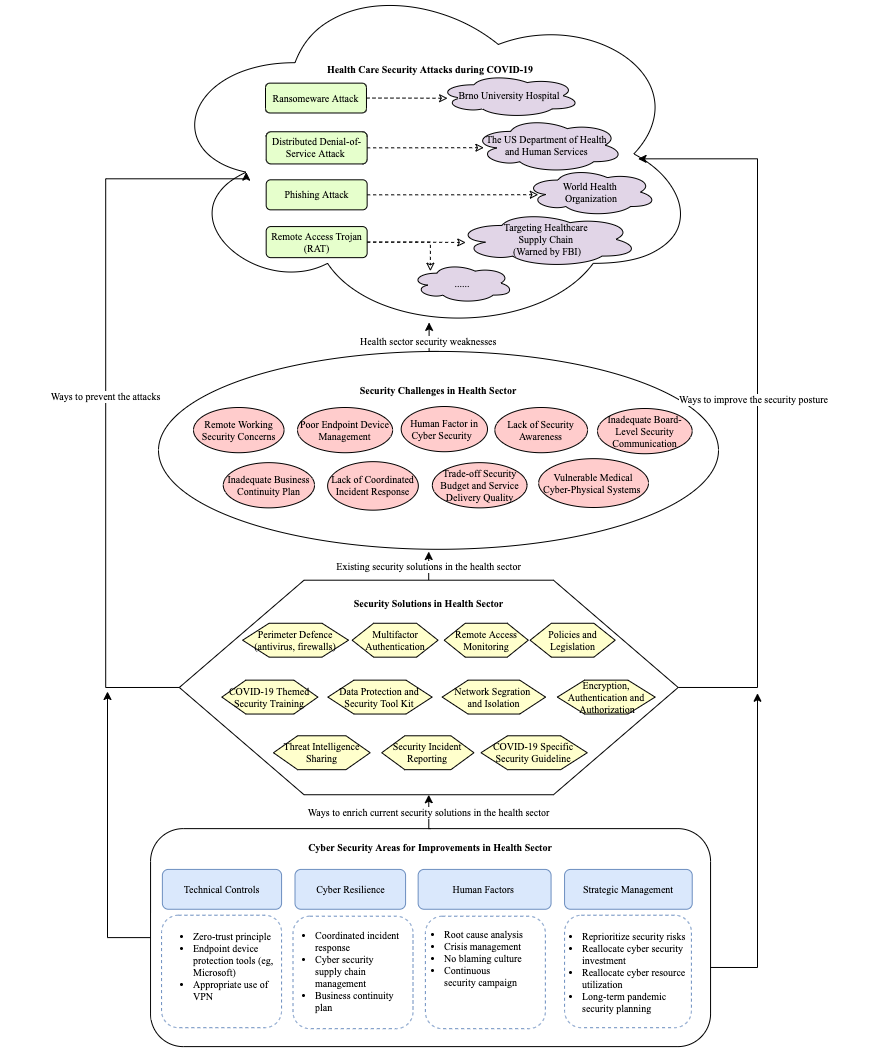
Security attacks, key security challenges, solutions, and areas to improve. FBI: Federal Bureau of Investigation; VPN: virtual private network.

Through a scoping review, this research identified key cybersecurity challenges, solutions adapted by the health sector, and areas to be improved in order to counteract the cyberattacks introduced through changes to working practices in the face of the COVID-19 pandemic. This review identified 9 main challenges in cybersecurity and 11 key solutions that health care organizations adapted to address these challenges. Based on our findings and analysis, we can conclude that the main challenges that the health sector faces due to the COVID-19 pandemic include increased reliance on remote working by staff, high demand for PPE by staff on the first line of defense, and decreased mobility due to the lockdown. Indeed, these changes have made the health sector vulnerable to potential cyberattacks. For example, remote work was taken up by users with little previous experience, and there was also no planning and cybersecurity-associated assurance prior to the shift. Furthermore, evidence can be seen from the security incidents that took place during the lockdown period such as those of Brno University Hospital, hospitals in Romania, etc. The health sector continues to face security challenges [1,17]. Challenges such as remote working security assurance, endpoint device management, inadequate business continuity plans, lack of security awareness, etc, are apparent in the health sector. There are some existing solutions employed by health care organizations, especially in the United Kingdom, such as remote access monitoring. Figure 2 summarizes the main findings from the literature review and highlights the gaps and vulnerabilities that were exploited during the cyberattacks that took place during the COVID-19 pandemic. However, there are still challenges and gaps to be addressed, as discussed below.

### Implications for Future Research

Although the health sector has made some efforts to address these challenges, more research is required in some domains.

#### Technical Controls

The health sector has applied some technical solutions to tackle cybersecurity challenges in order to secure the remote work environment and monitor endpoint applications. These include but are not limited to network security (eg, network segmentation), multifactor authentication, password protection, patching systems, and the use of intrusion detection and prevention systems. There are also innovative security solutions such as the zero-trust principle (ie, to treat all devices as untrustworthy before access or authorization can be considered). The use of VPNs is a popular technique in the remote work environment but is not always required. Health care organizations should avoid the abuse of VPNs and ensure it is applied to specific tasks, such as for system admin use and medical diagnosis purposes through access to legacy systems (eg, patient records management systems) stored on private data servers. Future research should explore innovative solutions such as blockchain as it can facilitate health care interoperability due to its immutability, transparency, and decentralization. In general, the health sector significantly lags behind other sectors in terms of cybersecurity. Future research should borrow experience from general cybersecurity practices (eg, NIST guidelines) and adapt them according to the needs of the health sector, especially in the context of pandemics.

#### Cyber Resilience

In order to improve system resilience, health organizations have some business continuity planning in place for data protection and recovery but lack a systematic way to maintain cyber resilience [18]. The vulnerabilities in the cyber supply chain makes it difficult to recover from an incident caused by third parties [38-41]. In the case of impact on medical devices or clinical information systems, incident response should be coordinated with device manufacturers and vendors. Health care organizations have realized the importance of having a comprehensive view of cybersecurity management in order to prevent cyberattacks [18] but have not built this coordinated capacity. There is a lack of a cyber resilience program to evaluate vendors’ capabilities around threat protection, particularly across email servers (phishing and ransomware), breadth of portfolio coverage in addressing cloud architecture, and endpoint security. Future research should focus on building a coordinated cybersecurity capacity in order to systematically assess vulnerabilities and respond to cyber threats.

#### Human Factors in Cybersecurity

People are likely to make mistakes, especially in the context of changes in their traditional way of working. Health care organizations are required to adopt a nonblaming culture in reporting incidents. The health sector should focus on root cause analysis [28] and prevent incidents from happening especially through unintentional human error. Published research has shown that the majority of information security incidents relate to human error [28,29], which is a vulnerability that attackers will look to exploit. A human error analytical approach such as IS-CHEC could be deployed both reactively, through integration within incident management practices [29,30], and proactively, through simple interaction with operational personnel [29], to detect current human error areas of weaknesses and apply associated remedial and preventative measures. Moreover, health care staff in the organization need to be educated and build awareness of the ongoing security situation during the COVID-19 pandemic. For example, in the case of infection, staff are required to disconnect from the network to contain the spread. Organizations should continuously raise awareness internally by launching campaigns even during a time of crisis (ie, to inform health staff not to open suspicious emails). Future research should focus on creating pandemic-themed security awareness campaigns. Moreover, a positive and empowering culture is also required (eg, by sharing the rate of people who did not click on phishing-negative emails during a training campaign). Experience can be borrowed from the organizational climate literature to positively influence people’s behavior [59].

#### Strategic Cybersecurity Management

Although health care organizations have invested in cybersecurity to counteraction security attacks, further efforts are needed to reprioritize cybersecurity risk assessment during the COVID-19 pandemic, reallocate security investment, and optimize resource utilization to obtain adequate assurances. According to Argwa et al [46], health care organizations are advised to allocate more resources and funding to cybersecurity. Strategic cybersecurity investment is still an immature research area in health care largely due to boards’ inability to fully understand and anticipate the direct and indirect impact on their health services. Further, there are language barriers between the technical team and the board [27]. Another reason is that the board finds it difficult to estimate the costs of investing and balancing these against potential benefits procured or impacts mitigated [8] as cybersecurity investments prevent potential losses but may not generate business benefits directly. Moreover, organizations should not only create security guidelines specific to the COVID-19 pandemic but also plan for the long term for remote working and spend efforts on strengthening their security mechanisms and cybersecurity crisis management capabilities. More research efforts are needed to support the top management teams of the health sector to understand the threat landscape and make better-informed decisions to allocate resources not just to provide services to staff and patients but also for protection and resilience, in order to continuously serve even in times of emergency such as the current pandemic and beyond.

### Limitations

Contrary to systematic reviews, scoping reviews are used to identify knowledge gaps, scope a body of literature, and clarify concepts. However, some limitations should be considered. Scoping reviews usually provide descriptive information in order to address the objectives of the review, which often leads to less defined searches. This review mitigated this limitation by clearly defining the search terms and search formula. Scoping reviews are also at risk of bias from different sources. All 4 authors were involved in the article identification, selection, and analysis processes in order to reduce the risks of bias. Because of variability when conducting a scoping review, there is a need for methodological standardization to ensure the strength of evidence. This review followed the PRISMA-ScR to standardize the process and improve the strength of evidence. Another limitation is that this review included exact terms used to search the titles or abstracts of existing publications. Any articles that used different terms, (eg, “computer security”) would not have been included. In addition, publications that were not written in English were excluded. Moreover, although this scoping review focused on health care, the solutions identified could be applied to other industries.

### Conclusions

The COVID-19 pandemic has challenged the resilience of the health care information system. This research was motivated by the urgency of counteracting the cyberattacks that have recently happened to hospitals, pharmaceutical companies, the US Department of Health and Human Services, and the WHO and its partners, etc. We performed a review on security challenges of the health sector and the solutions employed during COVID-19. We identified the root causes of the security incidents that have impacted the health sector during the COVID-19 pandemic, cybersecurity challenges, solutions, and areas in need of improvement. The results show that the main root causes of the security incidents that happened during the COVID-19 pandemic are mainly from phishing, ransomware, DDoS attacks, and malware. The main challenges faced by health care organizations are inadequate endpoint device management, lack of security awareness, insecure remote work environment, inadequate business continuity plans, lack of coordinated incident response, and difficulty in trading off security investment and service delivery quality. Needless to say, another major challenge is human error, both from the perspective of the health care worker at the frontline and those working from home. As the COVID-19 pandemic has shifted our priorities, there is a greater tendency for human error to occur when staff are preoccupied with saving lives, working in a strange or different environment, and using new or various technologies. With little or no experience and a lack of prior planning and training to work in such situations, health care workers require more than training and support, such as adequate time, documented procedures, and guidance on revised procedures and technology.

Although the health sector has made some efforts to address these challenges by applying technical measures, raising security awareness, enforcing policies, and developing COVID-19–specific guidelines, more research efforts are still required in some domains. Future research should focus on exploring enhanced technical controls through the adaption of general cybersecurity practices (eg, NIST guidelines); improving cyber resilience by building a coordinated cybersecurity capacity to systematically assess vulnerabilities of the complex health care supply chain and respond to cyber threats; reducing human-related security incidents by exploring human error reduction approaches and pandemic-themed awareness campaigns; and enhancing strategic cybersecurity management by exploring crisis management planning, security risks reprioritization, and the optimization of cybersecurity budget and resource reallocation.

Many health care organizations are applying a temporary solution to counteract cyber threats during the COVID-19 pandemic. These organizations should plan for the long term, provide adequate levels of cybersecurity resources to deal with fast-changing situations, and offer the required assurance within these changes. This paper provides useful insights for the health sector on their cybersecurity issues during the COVID-19 pandemic or other epidemic or pandemic situations in the future. Moreover, cybersecurity experience in other sectors can be borrowed and applied in the health sector.

## Appendix

Multimedia Appendix 1 PRISMA-ScR checklist.

## Abbreviations

DDoS: distributed denial of service
FBI: Federal Bureau of Investigation
IoT: internet of things
IT: information technology
NCSC: National Cyber Security Centre
NHS: National Health Service
NIST: National Institute of Standards and Technology
PPE: personal protective equipment
PRISMA-ScR: Preferred Reporting Items for Systematic Reviews and Meta-Analyses Extension for Scoping Reviews
VPN: virtual private network
WHO: World Health Organization
