# Correction: Health Care Cybersecurity Challenges and Solutions Under the Climate of COVID-19: Scoping Review

**DOI:** 10.2196/29877

**Published:** 2021-04-28

**Authors:** Ying He, Aliyu Aliyu, Mark Evans, Cunjin Luo

**Affiliations:** 1 School of Computer Science University of Nottingham Nottingham United Kingdom; 2 School of Computer Science and Informatics De Montfort University Leicester United Kingdom; 3 School of Computer Science and Electronic Engineering University of Essex Colchester United Kingdom; 4 Key Lab of Medical Electrophysiology, Ministry of Education Institute of Cardiovascular Research Southwest Medical University Luzhou China

In “Health Care Cybersecurity Challenges and Solutions Under the Climate of COVID-19: Scoping Review” (J Med Internet Res 2021;23(4):e21747) the authors noted one error.

In the originally published manuscript, a footnote was erroneously displayed in the authorship list noting equal contribution only for author Cunjin Luo.

This footnote has been removed from the corrected manuscript, as no equal contribution should be noted in the authorship list.

The correction will appear in the online version of the paper on the JMIR Publications website on April 28, 2021, together with the publication of this correction notice. Because this was made after submission to PubMed, PubMed Central, and other full-text repositories, the corrected article has also been resubmitted to those repositories.

