# Supplementary material for: Health Care Cybersecurity Challenges and Solutions Under the Climate of COVID-19: Scoping Review
Source: J Med Internet Res. 2021 Apr 20;23(4):e21747. doi: 10.2196/21747 (PMC8059789; doi:10.2196/21747)
Supplement: Multimedia Appendix 1 [file jmir_v23i4e21747_app1.docx]

**Appendix: PRISMA-ScR CHECKLIST ITEM**

| **SECTION** | **ITEM** | **PRISMA-ScR CHECKLIST ITEM** |
| --- | --- | --- |
| TITLE | | |
| Title | 1 | Healthcare Cyber Security Challenges and Solutions Under the Climate of COVID19: A Scoping Review |
| ABSTRACT | | |
| Structured  summary | 2 | Abstract provided a structured summary that includes background, objectives, methods, results, and conclusions that relate to the review questions and objectives. |
| INTRODUCTION | | |
| Rationale | 3 | The COVID19 pandemic has created a new reality for the health sector. The sector has become a primary target of adapted cybersecurity attacks. There are some research efforts reviewing the literature in cyber security in health sector. However, there are limited research efforts on an in-depth review and analysis of the key cyber security challenges and solutions in health sector specifically under the pandemic situation such as COVID19. |
| Objectives | 4 | This paper aims to identify the most prominent and significant methods of attack and threats that have affected the health sector during the COVID19 pandemic, the cybersecurity challenges, solutions as well as the areas that require further efforts in the community. |
| METHODS | | |
| Protocol and registration | 5 | This research follows the PRISMA-ScR protocol. The aim of this review is to identify health sector cyber-attacks, security challenges and solutions. Before undertaking this review, a protocol was created detailing the information sources, searching strategies, eligibility criteria, selection of sources of evidence and data charting processes. |
| Eligibility criteria | 6 | Articles in English in the last decade were included, i.e., 2011-2020. Reports, news articles, or websites were also included only when they are related directly to previously published work, or they were the only currently available information source at the moment of manuscript preparation. |
| Information sources | 7 | PubMed and Scopus |
| Search | 8 | The search formular “(covid or healthcare) and cybersecurity” was used to search for the articles. |
| Selection of sources of evidence | 9 | The selection process was illustrated in Figure 1. The results of search were exported to the Endnote library. The title and abstract of each paper were analysed by two of the authors to assess whether it fell into the specified criteria. In cases in which this was not obvious, all four authors looked at the paper and, when necessary, read it to assess relevance. |
| Data charting process | 10 | The data was extracted and stored in a standardized Microsoft Excel form. This was an iterative process whereby the charting table is continually updated. Data charting was carried out both independently and in duplicate by at least two authors to ensure the quality of the extracted key findings from the literature, before being used for results analysis. |
| Data items | 11 | Key data items including title, abstract, authorship, aims, key findings related to the review objectives, evidence document, document type, year of publication, and the location. |
| Critical appraisal  within sources of  evidence | 12 | Although JBI suggested that the critical appraisal is usually not needed for a scoping review, we had at least two authors checked the source of evidence, making sure that they are relevant, up to date and are from reputable sources. In cases in which this was not obvious, all four authors looked at the sources and assessed them. |
| Synthesis of results | 13 | Through aggregating the information from the selected literature, the results were analysed qualitatively (grouped into themes and presented in tables), reported in the Results section. |
| RESULTS | | |
| Selection of sources of evidence | 14 | As illustrated in Figure 1. 307 identified papers in total were screened. There were 53 duplicates removed, 57 papers excluded due to the lack of “healthcare or covid” core or no “cybersecurity” core in the abstract. A further 197 papers were excluded due to the lack of “healthcare or covid” core or no “cybersecurity” core in the full text. 56 papers were finally included in the review. |
| Characteristics of sources of evidence | 15 | For each source of evidence, relevant data with citation was grouped into four major themes and was presented in Table 1-4. |
| Critical appraisal  within sources of  evidence | 16 | As discussed in the method section, the source of evidence was checked by at least two authors to make sure that they are relevant, up to date and are from reputable sources. |
| Results of  individual sources  of evidence | 17 | For each included source of evidence, relevant data that related to the review questions and objectives, was charted and grouped into four themes and was presented in Table 1-4. |
| Synthesis of results | 18 | The charting results related to the review questions and objectives were summarised and presented in themes and in tables. |
| DISCUSSION | | |
| Summary of evidence | 19 | This section provided a summary of the key research results including cyber security challenges, cyber security solutions adopted by the health sector and the areas to be improved in order to counteract the cyber-attacks introduced through changes to working practices dealing with the current COVID19 pandemic, linking to the review questions and objectives. |
| Implications for future research |  | This section provided a summary of the key areas that requires more research efforts in the future, including technical controls, cyber resilience, human factors in cyber security, and strategic cyber security management. |
| Limitations | 20 | Scoping reviews are at risk for bias from different sources. This review followed the PRISMA-ScR to standardize the process and improve the strength of evidence. This review sincluded the exact terms used for searching in the titles or abstracts of existing publications. Any articles that used different terms would not have been included. |
| Conclusions | 21 | This section provided a summary of the interpretation of the results with respect to the review questions and objectives as well as the research areas to be improved in the future. |
| FUNDING | | |
| Funding | 22 | This work was supported by the National Natural Science Foundation of China (Grant No. 61803318). |
